# First isolation of La Crosse virus from naturally infected Aedes albopictus.

**DOI:** 10.3201/eid0705.017506

**Published:** 2001

**Authors:** R R Gerhardt, K L Gottfried, C S Apperson, B S Davis, P C Erwin, A B Smith, N A Panella, E E Powell, R S Nasci

**Affiliations:** University of Tennessee, Knoxville, USA.

## Abstract

La Crosse (LAC) virus, a California serogroup bunyavirus, is the leading cause of pediatric arboviral encephalitis in the United States and an emerging disease in Tennessee, West Virginia, and North Carolina. Human cases of LAC encephalitis in Tennessee and North Carolina have increased above endemic levels during 1997 to 1999 and may represent an expansion of a new southeastern endemic focus. This report describes the isolation of LAC virus from the exotic mosquito Aedes albopictus. The discovery of LAC virus in wild populations of Ae. albopictus coupled with its expanding distribution in the southeastern United States, suggests that this mosquito may become an important accessory vector, potentially increasing the number of human cases in endemic foci or expanding the range of the disease.


### First Isolation of *La Crosse Virus* from Naturally Infected *Aedes albopictus*

**Reid R. Gerhardt,* Kristy L. Gottfried,† Charles S. Apperson,‡ Brent S. Davis,† Paul C. Erwin,§ A. Brent Smith,* Nicholas A. Panella,† Eugene E. Powell,‡ and Roger S. Nasci†**

*University of Tennessee, Knoxville, Tennessee, USA; †Centers for Disease Control and Prevention, Fort Collins, Colorado, USA; ‡North Carolina State University, Raleigh, North Carolina, USA; and §State of Tennessee Department of Health, East Tennessee Region, Knoxville, Tennessee, USA

*La Crosse virus* (LAC), a California serogroup bunyavirus, is the leading cause of pediatric arboviral encephalitis in the United States and an emerging disease in Tennessee, West Virginia, and North Carolina. Human cases of LAC encephalitis in Tennessee and North Carolina have increased above endemic levels during 1997 to 1999 and may represent an expansion of a new southeastern endemic focus. This report describes the isolation of LAC virus from the exotic mosquito *Aedes albopictus.* The discovery of LAC virus in wild populations of *Ae. albopictus*, coupled with its expanding distribution in the southeastern United States, suggests that this mosquito may become an important accessory vector, potentially increasing the number of human cases in endemic foci or expanding the range of the disease.

Historically, *La Crosse virus* (LAC) encephalitis has been most common in the upper midwestern United States, primarily Illinois, Iowa, Indiana, Minnesota, Ohio, and Wisconsin (1), where the primary vector is *Ochlerotatus triseriatus*, a native mosquito that breeds in tree holes and artificial containers. In recent years, LAC encephalitis activity has increased in West Virginia, which has had more recorded cases than any other state, accounting for more than half of cases reported from 1996 to 1999 (2). A recent cluster of new cases in Tennessee is the first report of a possible extension of the West Virginia endemic focus and may represent a new southeastern endemic focus (1).

From 1963 to 1996, nine cases of pediatric LAC encephalitis were reported in Tennessee (1). From 1997 to 1999, however, the East Tennessee Regional Health Office in Knoxville confirmed 26 pediatric LAC encephalitis cases in Tennessee and southeastern Kentucky (10 confirmed cases in 1997 [1], 10 in 1998, and 6 in 1999 [3]) making LAC encephalitis the most common arboviral disease in Tennessee. During 1997 and 1999, counties reporting confirmed cases were Knox (9 cases); Anderson (3 cases); Cumberland (3 cases); Sevier and Claiborne (2 cases each); and Blount, Jefferson, Cocke, and Campbell (1 case each) in Tennessee; Bell County (1 case) in southeastern Kentucky; and 2 cases in the region with undetermined infection location (Erwin PC, unpub. data). The presumptive sites of transmission in Tennessee are in the Ridge and Valley Province, with the exception of three 1999 cases in Cumberland County, on the Cumberland Plateau. Despite the marked increase in human cases since 1996, LAC virus had never been isolated from any mosquito species in Tennessee before this finding. In a retrospective serologic study, the overall rate of seropositivity to LAC virus was 0.5% for human sera (n = 1,000) collected in 15 eastern Tennessee counties (3).

In contrast to Tennessee, approximately three cases per year of LAC encephalitis have been reported from western North Carolina from 1977 to 1995 (4,5). LAC virus has been isolated from *Oc. triseriatus* (6,7) collected from the homes of LAC encephalitis patients in North Carolina. As in Tennessee, LAC encephalitis cases have increased in North Carolina since 1996. Twenty-five cases were reported for the period 1996 to 1999, with 10 cases for 1999 and 5 cases for each of the other years (NC Department of Health and Human Services, unpub. data). In a retrospective serologic survey, the overall rate of seropositivity to LAC virus was 9.6% for human sera (n = 1,016) collected in 12 western North Carolina counties (5).

*Ae. albopictus*, a mosquito that breeds in tree holes and artificial containers, was discovered in Houston, Texas, in 1985. It was probably introduced from its natural range in Asia to the United States in imported used tire casings (8). Subsequently, this species has spread rapidly throughout much of the United States (8) and is now found in 928 counties and 30 states (Moore CG, pers. comm.), including all Tennessee (9) and North Carolina counties (Apperson CS, Harrison BA, unpub. data). In addition to being a nuisance, this species is known to transmit dengue, yellow fever, and a variety of other arboviruses. *Ae. albopictus* has been tested frequently for the presence of human arboviruses since its introduction into the United States (8). Eastern equine encephalitis virus, Cache Valley virus (8), and Jamestown Canyon virus (10) are the only human pathogens that have previously been isolated from naturally infected *Ae. albopictus* in the United States, although none of these isolations have been in association with an outbreak or known human cases. *Ae. albopictus* is a competent laboratory vector of LAC

Address for correspondence: Kristy L. Gottfried, Centers for Disease Control and Prevention, P.O. Box 2087, Fort Collins, CO 80522, USA; fax: 970-221-6476; e-mail: kig4@cdc.gov virus (11) and can transmit the virus vertically by the transovarial route (12,13).

Active surveillance for human LAC encephalitis cases and virus-infected mosquitoes has been conducted in eastern Tennessee since 1997. In North Carolina, case surveillance remains passive; however, efforts to collect virus-infected mosquitoes on the home properties of some case patients were made from 1997 to 1999. We describe the results of surveillance programs designed to examine container-breeding mosquitoes near the homes of LAC encephalitis patients in an attempt to isolate LAC virus from its vector(s) in eastern Tennessee and western North Carolina.

### Materials and Methods

### Collection Sites and Mosquito Rearing

During 1997 to 1999 in Tennessee, surveillance of container-breeding mosquitoes was conducted weekly at the homes of six patients with confirmed LAC encephalitis. Collections were made from two sites in Knox County (Karns and Oak Ridge Highway) and Anderson County (Clinton and Holt Road) and one site each in Blount County (Townsend) and Cumberland County (Crab Orchard) (Figure 1). In North Carolina, mosquitoes were collected weekly at nine sites on the Cherokee Indian Reservation (six in Jackson and three in Swain counties) and 10 sites in Buncombe County (four in 1998 and six in 1999) (Figure 1). Except for one site in Buncombe County, all sites were the homes of LAC encephalitis patients. Standard oviposition traps (14) with seed germination paper as an oviposition substrate (15,16) were used to collect eggs of *Ae. albopictus* and *Oc. triseriatus*. In Tennessee, 30 oviposition traps were placed at Karns, 20 at Oak Ridge Highway, and 10 at each of the other sites. In North Carolina during 1998, four oviposition traps were placed at each site, and in 1999, eggs were collected from 10 oviposition traps per site.

Eggs on the oviposition strips were hatched by placing them in 2 L $H_2O$ with 2.5 g of liver powder or a mixture of liver powder:brewers yeast (1:2 by weight) for 48 hours. Larvae were reared at 28°±2°C with constant light. Immediately after adult emergence, the mosquitoes were sorted by species and sex, placed in pools of $\leq$50 individuals, and stored at 70°C until tested for the presence of virus.

### Virus Isolation and Identification

Pools of mosquitoes were placed in 12 x 75 mm (5 mL) Falcon tubes (Becton Dickinson Labware, Franklin Lakes, NJ) with 2 mL BA-1 diluent (1 x M199 with Hanks balanced salt solution, 0.05 M Tris buffer [pH 7.6], 1% bovine serum albumin, 0.35 g/L sodium bicarbonate, 100 g/L streptomycin, 100 units/mL penicillin, and 1 g/mL Fungizone [Apothecon, Princeton, NJ]). Pooled mosquitoes were ground by placing four 4.5-mm steel beads (BB caliber airgun shot) into the tube with the mosquitoes and diluent and vortexing for 20 to 30 seconds. The mosquito homogenate and steel beads were centrifuged to form a pellet.

Specimens were tested for the presence of live virus with a Vero cell culture plaque assay in six-well plates (17). Supernatant from the centrifuged mosquito homogenate (0.1 mL) was added to each of two wells, incubated at 37°C for 60 minutes, and covered with 3.0 mL of an agar overlay (1% Seakem LE agarose in M199, 0.2% sodium bicarbonate, 100 units/mL penicillin, 100 µg/mL streptomycin, 250 µg/mL gentamycin, and 4.5 µg/mL Fungizone). After 4 days of incubation (37°C, 4.7% $CO_2$), 3.0 mL of a second 1% agar overlay containing 0.004% neutral red was added to each well, and incubation was continued. Wells were examined for plaques daily for 10 days. Cells in virus-positive wells were harvested in 2 mL BA-1 diluent containing 20% fetal calf serum and frozen at -70°C. The positive original mosquito homogenates were reinoculated on Vero cells to confirm the presence of virus.

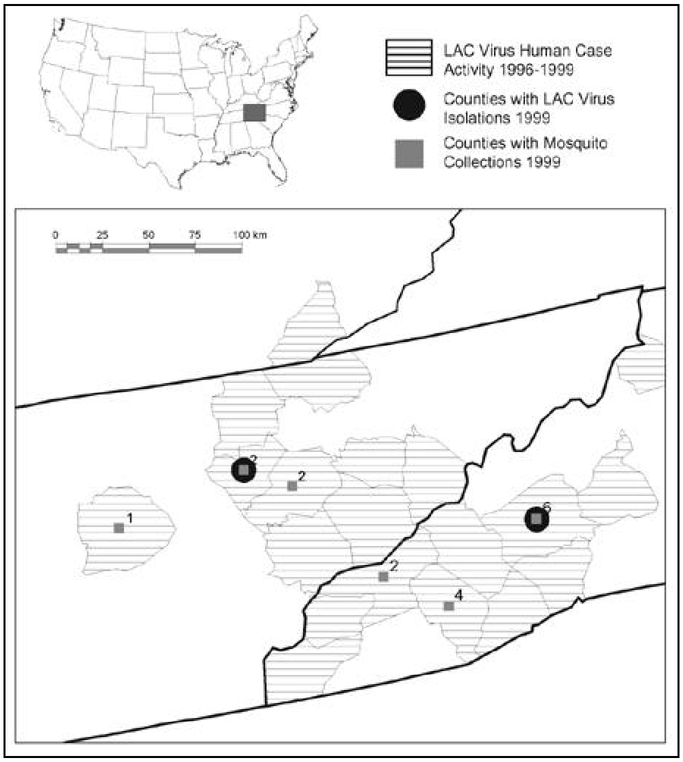

Figure 1. Regional map of eastern Tennessee, western North Carolina, and southeastern Kentucky, showing counties reporting human cases of La Crosse encephalitis from 1996 to 1999, counties with 1999 mosquito collection sites, and counties with 1999 *La Crosse virus* isolations from *Aedes albopictus*.

Virus isolates were identified by reverse transcription polymerase chain reaction (RT-PCR), followed by genomic sequencing performed on an ABI Prism 377 Sequencer (Applied Biosystems/PerkinElmer, Foster City, CA) according to the manufacturer's recommendations. Previously published S-segment (BCS82C, BCS332V) and L-segment (LCL80C, LCL199V) (18) and LAC M-segment primers (5'-CTTCATATTGACCACATG-3', 5'-CCATGCCTGTTTCAAT-CAGCATATGTC-3') were used to amplify viral RNA by RT-PCR for sequencing.

### Molecular Identification of the Species in the Original Mosquito Pool

Genomic DNA was extracted from the original ground mosquito pool. The homogenate (50 µL) was incubated at 65°C for 30 minutes in a 1.7-mL microcentrifuge tube (Co-Star, Corning, NY). Potassium acetate (7 µL of 8 M) was added for a homogenate final concentration of 1 M, incubated on ice for 30 minutes, and centrifuged for 15 minutes at 14,000 rpm. The supernatant was transferred to a fresh 1.7-mL microcentrifuge tube. DNA was precipitated by adding 140 µL of absolute ethanol, mixing by inverting the tube gently, and incubating at room temperature for 5 minutes. After centrifugation at 14,000 rpm for 15 minutes, the pellet was washed with cold 70% ethanol and resuspended in 10 µL of 10 mM tris buffer solution (pH 8.5).

Species-specific primers were designed to amplify the large subunit ribosomal DNA sequence of *Ae. albopictus* and *Oc. triseriatus*. These primers were used to confirm the species composition of the original mosquito pool. The oligonucleotides were designed from the complete rDNA sequences published for *Oc. triseriatus* (19) and *Ae. albopictus* (20). The 18-bp forward primer (5'-CGTGGATCGATGAAGACC-3'), located in the highly conserved 5.8s region of rDNA, was used for both species. The reverse primers for both species were unique for that species and located in the ITS2 region of rDNA. The reverse primer for *Ae. albopictus* was 5'-GACACCGCACCGCACAACTCACAC-3' and *Oc. triseriatus* was 5'-TATGCTATCCGTTCGAGAG-3'. The Expand Fidelity PCR System (Roche Diagnostics, Indianapolis, IN) was used to amplify the DNA product. Each 50-µL PCR reaction contained 5 µL 10X buffer #2, 2 µL (10 µM) of forward and reverse primer, 1 µL (10 mM) deoxynucleoside-triphosphate (dNTP) mix, 5 µL genomic mosquito pool DNA, and 34 µL of nuclease-free water. Primer reactions were amplified in a Perkin-Elmer model 9600 thermocycler (GeneAmp PCR System). The reaction was incubated at 94°C for 5 minutes, followed by 4°C after 1 µL of enzyme was added. This 50-µL reaction was amplified by PCR for 1 cycle of 94°C for 15 seconds, 50°C for 20 seconds, and 68°C for 1 minute; 9 cycles of 94°C for 15 seconds, 55°C for 20 seconds, and 68°C for 1 minute; and 20 cycles of 94°C for 15 seconds, 55°C for 20 seconds, and 68°C for 1 minute + 5 seconds/cycle. The amplification product was analyzed by electrophoresis of 5 µL of the reaction on a 2% agarose gel containing ethidium bromide and visualized by UV light. The sensitivity and specificity of the species-specific primers were evaluated by using pools of *Ae. albopictus, Oc. triseriatus,* and *Ae. albopictus/Oc. triseriatus* combinations (Figure 2).

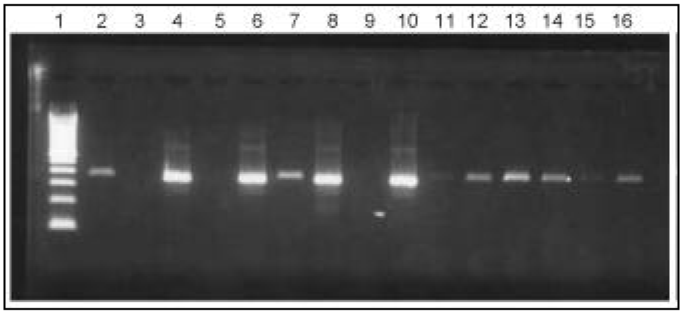

Figure 2. Species-specific gel electrophoresis of *La Crosse virus*-positive mosquito pools from Tennessee and North Carolina and *Aedes albopictus* pools with varying degrees of *Ochlerotatus triseriatus* contamination. Lane 1, 100-bp ladder; lane 2, *Oc. triseriatus* pool with *Oc. triseriatus* primers; lane 3, *Oc. triseriatus* pool with *Aedes albopictus* primers; lane 4, *Ae. albopictus* pool with *Ae. albopictus* primers; lane 5, *Ae. albopictus* pool with *Oc. triseriatus* primers; lane 6, *Ae. albopictus* pool contaminated by 1 *Oc. triseriatus* individual, with *Ae. albopictus* primers; lane 7, *Ae. albopictus* pool contaminated by 1 *Oc. triseriatus* individual, with *Oc. triseriatus* primers; lane 8, TN00-2266 *Ae. albopictus* pool with *Ae. albopictus* primers; lane 9, TN00-2266 *Ae. albopictus* pool with *Oc. triseriatus* primers; lane 10, NC00-1547 *Ae. albopictus* pool with *Ae. albopictus* primers; lane 11, NC00-1547 *Ae. albopictus* pool with *Oc. triseriatus* primers; lane 12, *Ae. albopictus* pool contaminated by 1 *Oc. triseriatus* head, with *Oc. triseriatus* primers; lane 13, *Ae. albopictus* pool contaminated by 1 *Oc. triseriatus* thorax, with *Oc. triseriatus* primers; lane 14, *Ae. albopictus* pool contaminated by 1 *Oc. triseriatus* abdomen, with *Oc. triseriatus* primers; lane 15, *Ae. albopictus* pool contaminated by 3 *Oc. triseriatus* legs, with *Oc. triseriatus* primers; and lane 16, *Ae. albopictus* pool contaminated by 6 *Oc. triseriatus* legs, with *Oc. triseriatus* primers.

### Results

For Tennessee, 10,717 mosquitoes were reared to the adult stage and analyzed for virus (Table). At all collection sites, the *Ae. albopictus* population density was approximately fourfold greater than that of *Oc. triseriatus*. LAC virus was isolated from one pool of 14 female *Ae. albopictus* (TN00-2266) collected at the Clinton site in Anderson County on August 16, 1999. When base sequences of the 120-bp L-segment, 775-bp M-segment, and 251-bp S-segment amplicons were compared with published LAC virus genome sequences (GenBank U-12396, U-18980, AF025479), homology was 98%, 95%, and 100%, respectively, confirming the product is LAC virus. By PCR analysis, the species composition of the original mosquito pool was *Ae. albopictus* only, with no detectable traces of *Oc. triseriatus* tissue (Figure 2). The minimum field infection rate (21) for *Ae. albopictus* oviposited during the week the positive pool was collected is estimated to be 6.5 infected mosquitoes per 1,000 specimens. The ratio of *Ae. albopictus* to *Oc. triseriatus* was 153:1 for the weekly collection (August 16, 1999) containing the positive mosquito pool. To ensure that no isolates were missed in the initial Vero cell plaque assay screening, all *Ae. albopictus* and *Oc. triseriatus* mosquito pools collected from the Clinton site during August were tested by RT-PCR for the presence of LAC viral RNA and found to be negative.

In North Carolina, LAC virus was isolated from one pool of 44 female *Ae. albopictus* (NC00-1547) and one pool of 50 male *Oc. triseriatus* collected on September 6, 1999, from the same site in Buncombe County. When base sequences of the 120-bp L-segment, 775-bp M-segment, and 251-bp S-segment amplicon were compared with published LAC virus genome sequences (GenBank U-12396, U18980, AF025479), homology was 96%, 95%, and 100%, respectively, confirming the product is LAC virus. Minimum field infection rates (21) for the week of the LAC virus-positive oviposition trap collections were 4.7 and 3.8 infected mosquitoes per 1,000 specimens for *Ae. albopictus* and *Oc. triseriatus*, respectively.

PCR analysis to verify the species composition of the original mosquito pool suggested that the *Ae. albopictus* pool was contaminated with *Oc. triseriatus* tissue (Figure 2). The putative *Oc. triseriatus* amplicon could not be sequenced because of the small amount of product initially amplified. However, in a subsequent PCR test, amplification for an extended period of time provided sufficient material. Sequence analysis of the amplicon matched the *Oc. triseriatus* sequence (19), verifying that the *Ae. albopictus* pool contained *Oc. triseriatus* tissue.

The Clinton site where the positive mosquito pool from Tennessee was obtained is the home of a child who had onset of LAC encephalitis on June 23, 1998. The residence is well maintained, with few permanent or disposable containers; the property is partially wooded, with oak and hickory trees. The house and property are within 15 m of an oak and hickory forest. Numerous disposable containers were observed along the road (10 m distant) and at a nearby residence (40 m). The Buncombe County residence where infected mosquitoes were collected is the site where a child is presumed to

Table. Numbers of *Aedes albopictus* and *Ochlerotatus triseriatus* adults reared from eggs and pooled specimens tested for *La Crosse virus* from mosquitoes collected at La Crosse encephalitis case sites in eastern Tennessee and western North Carolina, 1999

| | | *Ae. albopictus* | | *Oc. triseriatus* | |
|---|---|---|---|---|---|
| County-case site | Collection dates | No. individuals | No. pools | No. individuals | No. pools |
| Tennessee | | | | | |
| Knox-Karns | 5/10-9/18 | 3,999 | 131 | 1,838 | 72 |
| Knox-Oak Ridge Hwy | 8/10-9/17 | 464 | 16 | 10 | 2 |
| Anderson-Clinton[a] | 6/6-9/13 | 2,733 | 92[a] | 649 | 40 |
| Anderson-Holt Road | 8/10-9/17 | 309 | 11 | 36 | 2 |
| Blount-Townsend | 8/23-9/15 | 252 | 8 | 0 | 0 |
| Cumberland-Crab Orchard | 8/13-9/6 | 427 | 15 | 0 | 0 |
| Total | | 8,184 | 181 | 2,533 | 116 |
| North Carolina | | | | | |
| Buncombe-site 9[b] | 5/20-10/4 | 1,427 | 86 | 1,944 | 87 |

[a]Virus isolated from one pool of *Ae. albopictus* (14 females).
[b]Virus isolated from one pool of *Ae. albopictus* (44 females) and three pools of *Oc. triseriatus* (4, 6, and 50 males).

have contracted LAC encephalitis on July 30, 1997. The residence is one of numerous mobile homes located 50 m from the Swannanoa River. *Ae. albopictus* and *Oc. triseriatus* larvae have been periodically collected from discarded containers in a trash dump behind the mobile home park adjacent to the river. Most of this area is heavily shaded by mature oaks and poplars, and the understory is made up of knee-high grasses and other herbaceous plants.

### Conclusion

We report the first isolation of LAC virus from naturally infected *Ae. albopictus* mosquitoes (TN00-2266). The discovery of vertically infected *Ae. albopicus* in the field indicates that an adult female mosquito fed on a viremic host, became infected with the virus, and successfully transmitted the virus to offspring. However, the possibility that the adult female was infected by the venereal or transovarial route cannot be completely dismissed. In light of the ability of this species to orally transmit LAC virus (11), this finding indicates that *Ae. albopictus* may become an important accessory vector of LAC virus in enzootic foci and may facilitate expansion of the existing enzootic foci into new areas.

We believe that the North Carolina isolate from the *Ae. albopictus* pool resulted from a single virus-infected *Ae. albopictus*, rather than from contamination by a single virus-infected *Oc. triseriatus*. *Oc. triseriatus* tissue was present in the original mosquito pool that was amplified by PCR and visualized by gel electrophoresis (Figure 2). *Ae. albopictus* pools were made with known degrees of *Oc. triseriatus* tissue contamination (three and six legs, head, thorax, abdomen) (Figure 2). The original pool likely did not contain an intact *Oc. triseriatus* specimen because the amplification intensity was considerably lower than that produced by a whole mosquito (Figure 2). Instead, at least two *Oc. triseriatus* legs were likely present in the *Ae. albopictus* pool. However, the possibility of LAC virus contamination cannot be completely dismissed because an LAC virus isolate was obtained from *Oc. triseriatus* collected from the same site on the same date.

*Ae. albopictus* has been collected at every site examined (68 sites) in 13 Tennessee counties from 1997 to 1999 (Gerhardt RR, Gottfried KL, unpub. data). Although once considered rare in Tennessee, the species is now ubiquitous in the eastern part of the state. Likewise, in western North Carolina, *Ae. albopictus* is widely distributed throughout the mountains, where LAC virus transmission is endemic (6).

The relationship between the recent increase in LAC encephalitis cases (1997 to present) in the region and the expanding range of *Ae. albopictus* is one of association at this time. Additional research is needed to firmly establish *Ae. albopictus* as a vector of LAC virus in eastern Tennessee and western North Carolina. The remarkable disparity in seroprevalence rates of LAC antibodies in human sera between eastern Tennessee (0.5%) and western North Carolina (9.6%) provides indirect evidence that the disease is relatively new in the eastern Tennessee region. We can only speculate that the continued range expansion of *Ae. albopictus* will result in an increase in both incidence and distribution of LAC virus in Tennessee and North Carolina and throughout the southeastern United States.

### Acknowledgments

We thank Ken Tennessen, Chester G. Moore, Mary Crabtree, Sandy Halford, Tim F. Jones, and L.E.R. Patterson for their assistance.

This work was supported in part by contract T9006 from the North Carolina Department of Environment and Natural Resources to CSA and by financial support provided by the North Carolina Agricultural Research Service at North Carolina State University, the University of Tennessee Agricultural Experiment Station, the University of Tennessee (Knoxville), and the Tennessee Valley Authority Division of Resource Stewardship.

K. Gottfried's contribution to this publication was supported in part by an appointment to the Emerging Infectious Diseases Fellowship program administered by the Association of Public Health Laboratories and funded by the Centers for Disease Control and Prevention.

Dr. Gerhardt is a professor of entomology at the University of Tennessee, Knoxville. His research interests includes medical and veterinary entomology with emphasis in biology and ecology of mosquitoes, ticks, and biting flies.

### References

1. Jones TF, Craig AS, Nasci RS, Patterson LER, Erwin PC, Gerhardt RR, et al. Newly recognized focus of La Crosse encephalitis in Tennessee. Clin Infect Dis 1999;28:93-7.
2. Nasci RS, Moore CG, Biggerstaff BJ, Panella NA, Liu HQ, Karabatsos N, et al. La Crosse encephalitis virus habitat associations in Nicholas County, West Virginia. J Med Entomol 2000;37:559-70.
3. Jones TF, Erwin PC, Craig AS, Baker P, Touhey KE, Patterson LER, et al. Clinical evidence in children and serosurvey of La Crosse virus infections in Tennessee. Clin Infect Dis 2000;31:1284-7.
4. Kelsey DS, Smith B. California virus encephalitis in North Carolina. N C Med J 1978;39:654-6.
5. Szumlas DE, Apperson CS, Hartig PC, Francy DB, Karabatsos N. Seroepidemiology of La Crosse virus infection in humans in western North Carolina. Am J Trop Med Hyg 1996;54:332-7.
6. Kappus KD, Calisher CH, Baron RC, Davenport J, Francy DB, Williams RM. La Crosse virus infection and disease in western North Carolina. Am J Trop Med Hyg 1982;31:556-60.
7. Szumlas DE, Apperson CS, Powell EE, Hartig P, Francy DB, Karabatsos N. Relative abundance and species composition of mosquito populations (Diptera: Culicidae) in a La Crosse virus-endemic area in western North Carolina. J Med Entomol 1996;33:598-607.
8. Moore CG, Mitchell CJ. *Aedes albopictus* in the United States: Ten-year presence and public health implications. Emerg Infect Dis 1997;3:329-34.
9. Moore JP. *Aedes albopictus* (Diptera: Culicidae) occurrence throughout Tennessee, with biological notes. Entomol News 1998;109:363-5.
10. Gottfried KL, Gerhardt RR, Nasci RS, Karabatsos N, Crabtree MB, Burkhalter KL, et al. Temporal abundance, parity, survival rates and arbovirus isolation of field-collected container-inhabiting mosquitoes in eastern Tennessee. J Am Mosq Control Assoc. In press 2001.
11. Grimstad PR, Kobayashi JF, Zhang M, Craig GB Jr. Recently introduced *Aedes albopictus* in the United States: potential vector of La Crosse virus (Bunyaviridae: California serogroup). J Am Mosq Control Assoc 1989;5:422-7.
12. Tesh RB. Experimental studies on the transovarial transmission of Kunjin and San Angelo viruses in mosquitoes. Am J Trop Med Hyg 1980;29:657-66.
13. Tesh RB, Gubler DJ. Laboratory studies of transovarial transmission of La Crosse and other arboviruses by *Aedes albopictus* and *Culex fatigans*. Am J Trop Med Hyg 1975;24:876-80.
14. Loor KA, De Foliart GR. An oviposition trap for detecting the presence of *Aedes triseriatus* (Say). Mosq News 1969;29:487-8.
15. Szumlas DE, Apperson CS, Powell EE. Seasonal occurrence and abundance of *Aedes triseriatus* and other mosquitoes in a La Crosse virus-endemic area in western North Carolina. J Am Mosq Control Assoc 1996;12:184-93.
16. Steinley BA, Novak RJ, Webb DW. A new method for monitoring: mosquito oviposition in artificial and natural containers. J Am Mosq Control Assoc 1991;7:649-50.
17. Beaty BJ, Calisher CH, Shope RS. Arboviruses. In: Schmidt NJ, Emmons RW, editors. Diagnostic procedures for viral, rickettsial and chlamydia infections. Washington: American Public Health Association; 1989. p. 797-856.
18. Kuno G, Mitchell CJ, Chang GJ, Smith GC. Detecting bunyaviruses of the Bunyamwera and California serogroups by a PCR technique. J Clin Microbiol 1996;34:1184-8.
19. Reno HE, Vodkin MH, Novak RJ. Differentiation of *Aedes triseriatus* (Say) from *Aedes hendersoni* Cockerell (Diptera:Culicidae) by restriction fragment length polymorphisms of amplified ribosomal DNA. Am J Trop Med Hyg 2000;62:193-9.
20. Kjer KM, Baldridge GD, Fallon AM. Mosquito large subunit ribosomal RNA: simultaneous alignment of primary and secondary structure. Biochim Biophys Acta 1994;1217:147-55.
21. Nasci RS, Mitchell CJ. Arbovirus titer variation in field collected mosquitoes. J Am Mosq Control Assoc 1996;12:167-71.
